# Nanoliposomes and Nanoemulsions Based on Chia Seed Lipids: Preparation and Characterization

**DOI:** 10.3390/ijms21239079

**Published:** 2020-11-29

**Authors:** Daria V. Kuznetcova, Michel Linder, Carole Jeandel, Cedric Paris, Frederic Desor, Denis A. Baranenko, Liudmila A. Nadtochii, Elmira Arab-Tehrany, Frances T. Yen

**Affiliations:** 1 LIBio, ENSAIA-Université de Lorraine, 54505 Vandoeuvre-les-Nancy CEDEX, France; michel.linder@univ-lorraine.fr (M.L.); carole.jeandel@univ-lorraine.fr (C.J.); cedric.paris@univ-lorraine.fr (C.P.); 2 UR AFPA, ENSAIA-Université de Lorraine, 54505 Vandoeuvre-les-Nancy CEDEX, France; frederic.desor@univ-lorraine.fr; 3 Faculty of Biotechnologies, ITMO University, 191002 Saint-Petersburg, Russia; denis.baranenko@itmo.ru (D.A.B.); l_tochka@itmo.ru (L.A.N.)

**Keywords:** chia seeds, PUFA, nanoliposome, nanoemulsion

## Abstract

Polyunsaturated fatty acids (PUFA) are important in reducing the risk for cardiovascular, metabolic and neurodegenerative diseases. Chia (*Salvia hispanica* L.) seeds contain high levels of omega-3 PUFA, α-linolenic acid (ALA) in particular, and are a potential source for development of omega-3 PUFA-based products. Our objective was to obtain and characterize chia seed lipids, focusing on phospholipid fraction, and to investigate their use in the formulation of nanoemulsions (NE) and nanoliposomes (NL). Solvent-based lipid extraction was performed on the ORURO variety of chia seeds, followed by lipid composition analysis using GC and LC-MS and physico-chemical characterization of chia NL and NE. Folch extraction led to a slightly higher yield of ALA as compared to Soxhlet extraction. Lipid, phospholipid, and fatty acid composition analysis of the oil and residue revealed that the residue was rich in phospholipids; these were used to prepare NE and NL. Physico-chemical characterization showed that NE and NL were generally spherical (transmission electron microscopy), with a size of <120 nm under hydrated conditions that remained stable over 5 days. In conclusion, chia oil and phospholipid-rich residue can be used to obtain stable NL or NE using a simple method that involves spontaneous emulsification during lipid hydration, which potentially may be useful in cosmetics, pharmaceutical, and other health applications.

## 1. Introduction

Omega-3 polyunsaturated fatty acids (PUFA) are important in nutrition and health, playing an integral part in lipid homeostasis by virtue of their functions in modulating cell membrane fluidity and function, in cell signaling, and in the transcriptional regulation of gene expression. Numerous studies have demonstrated the relationship between PUFA deficiencies and risk for diseases including metabolic syndrome, insulin resistance, cardiovascular and neurodegenerative diseases [1,2]. Investigations have focused on their use in both preventive, as nutritional supplements, as well as in curative strategies as potential therapeutics of these lipid-related disorders. Omega-3 fatty acids are found in both marine organisms and plants. Because the former can be limited in supply or present a problem due to its allergenicity, interest has become focused on plant-based products as an interesting alternative and promising source of PUFA [3]. Seed oils are particularly rich in α-linolenic acid (ALA), which is an essential fatty acid and precursor of docosahexaenoic acid (DHA) and eicosapentaenoic acid (EPA). ALA itself also displays anti-inflammatory and neuroprotective properties, and therefore is of potential interest as a risk-reducing agent of related diseases.

Chia (*Salvia hispanica* L.) seeds are the richest ALA plant source known today. This plant was originally native to southern Mexico and northern Guatemala, and is now cultivated all over the world, including in Europe. Both the seeds and oil are EU-approved for consumption [4,5,6], and studies have highlighted their prophylactic properties for metabolic syndrome, hypertension, and cardiovascular diseases. Studies of the composition of chia seeds revealed a content of around 65% ALA of total fat, and up to 20% of linoleic acid [2,7,8,9,10]. In addition, chia seed oil also contains antioxidants [2,7,10,11,12], which could also contribute to the interest of this product in nutrition and health.

However, the high PUFA levels in chia seed oil (about 80%) also make it prone to oxidation which could lead to a loss of nutritional value during storage, as well as the possible formation of undesirable toxic products. The oxidative stability is the most important indicator of edible vegetable oil quality [2,9]. Thus, improving the stability of chia seed oil and preserving PUFA from degradation is of particular importance for its use in nutrition and health applications, such as functional foods or in drug development.

The use of nanotechnology makes it possible to change the traditional characteristics of edible oils, improve their quality and safety, increase bioavailability, and expand the scope of applications [13]. Nanoemulsions prepared from oils are of particular interest for encapsulation, preservation, and elaboration of nanocarriers of sensitive biologically active compounds. The properties of delivery systems based on nanoemulsions depend on the surfactants that coat the lipid droplets and are simple to manufacture, displaying high bioavailability due to particle size, as well as gravitational stability [14,15]. However, conventional nanoemulsions are limited as a potential drug delivery system in that only lipophilic molecules can be encapsulated, which significantly limits its use. This can be overcome by the use of nanoliposomes, which by virtue of their structural morphology, permit the encapsulation or incorporation of both hydrophilic and lipophilic molecules. This immediately widens the range of their applications for use in functional foods, cosmetics, and pharmaceuticals. Nanosized liposomes are multilamellar and have a large surface area, thus increased potential for solubility, bioavailability, improved controlled release, and more accurate targeting [16]. We have, in our laboratory, developed the technology enabling the preparation of PUFA-rich nanoliposomes prepared from phospholipid rich extracts, and have recently shown neurotrophic effects of nanoliposomes prepared from lecithin-rich extracts of fish by-products [17] that can be used as vectors for the delivery of anti-inflammatory drugs such as curcumin [18].

In view of the interest of PUFA-rich chia seeds and their bioactivities, our goal in this study was to obtain and characterize chia seed lipids, focusing on phospholipid fraction, and to investigate their use in the formulation of nanoemulsions (NE) and nanoliposomes (NL). Studies have reported the use of chia seed lipid fraction for the formulation of microemulsions using various materials as emulsifier and different microcapsule wall techniques [19,20,21,22,23,24,25,26], but little information is available on the use of ALA-rich chia seed lipids in nanosized particles [15,27]. Here, we demonstrate the feasibility of using chia seed lipids for preparing stable NL and NE, nanocarriers that have potential uses in both prevention and therapeutic applications.

## 2. Results

### 2.1. Chia Seed Lipid Extraction

For this study, we selected the chia ORURO variety, which has been specifically adapted for cultivation in Europe. Since oil yield and fatty acid composition of plants is variety-dependent [28,29,30], our first goal was to assess these parameters in the oil extracted from these seeds.

Lipid extraction by modified Folch method was chosen as the target extraction method to be used for chia seeds. This method uses a mixture of chloroform:methanol (2:1, *v/v*) which allows the extraction of polar lipids, including phospholipids for the preparation of nanoliposomes. Oil yield was found to be 29.37 ± 0.97% (*n* = 10) of total seed mass. This was 17% lower than that obtained when compared to the method using the Soxhlet apparatus and a diethyl ether/petroleum ether mixture (1:1), which led to an oil yield of 35.25 ± 1.42% (*n* = 5, *p* < 0.05) of total seed mass.

We compared oil yield obtained after Folch extraction of chia and two other seed types, flax and rapeseed, known to be rich in unsaturated fatty acids. Oil yield from chia seeds using the Folch-based method was significantly lower as compared to that of flax seed (38.63 ± 1.33%, *n* = 3, *p* < 0.05) or rapeseed (42.83 ± 1.33%, *n* = 3, *p* < 0.05); the oil yields of flax seed and of rapeseed were not statistically significantly different.

### 2.2. Characterization of Chia Seed Lipid Fraction

#### 2.2.1. Fatty Acid Composition and Characterization of Lipid Classes

Fatty acid (FA) composition of the lipid fraction of chia seeds was next analyzed using gas chromatography after extraction using the Soxhlet and Folch-based methods (Table 1).

Analysis of the chromatograms revealed 10 FA species, with the highest levels being ALA, linoleic, palmitic, oleic and stearic acids, in descending order. For the Folch method, there was a small but significantly lower amount of palmitic acid. On the other hand, % ALA was slightly but significantly higher with the Folch as compared with the Soxhlet methods. Analysis of polar lipid content of oil by thin-layer chromatography (Iatroscan) revealed no significant difference between that obtained after Folch (2.21 ± 0.33%, *n* = 6) or Soxhlet extraction (2.34 ± 0.61%, *n* = 3).

The evaporation step of the solvent after lipid extraction by the Folch-based method revealed the presence of a solid residue on the walls of the flask, which could not be completely re-dissolved in chloroform:methanol (2:1, *v/v*). This represented a yield of 13.97 ± 0.25% (*n* = 5) of oil mass, and we hypothesized that a complex, including phospholipids as heavy phase, had formed with other components of the chia seeds during the evaporation step. To test this, the composition of this solid residue obtained was analyzed, and found to contain moisture (10.13 ± 2.6%), protein (3.59 ± 0.15%), ash (2.61 ± 0.56%), dietary fiber (1.79%) relative to the total mass. Results showed that free sugars (glucose and fructose) were only a minor component (< 0.05%).

Analyses of the oil phase obtained by the modified Folch method were also carried out before and after solvent evaporation to determine if this step could affect the recovery of polar lipids. The results in Table 2 showed significant decreases in polar lipid content after solvent removal for the chia and rapeseed oil fractions. Interestingly, solid residue was found on the wall of the flask for all seed types. Polar lipid content was not statistically significant difference between the different seed types, either before or after the solvent evaporation step.

**Figure 1:**
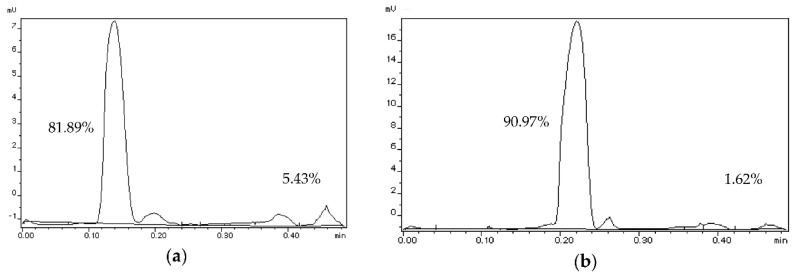
Separation for lipid classes by IATROSCAN: (**a**) Chia lipids before solvent removal; (**b**) Chia lipids after solvent removal.

An example of this is shown in Figure 1, following Folch extraction of chia seeds. Polar lipid content of the lipid extract before evaporation of the solvent was decreased two-fold after evaporation and solid residue formation.

Based on these analyses, we would therefore propose that this solid residue consists of a polar lipid-rich natural complex containing moisture, protein and fiber, and other components as described above, which could explain the significant loss of the polar lipid content in the oily phase following evaporation after Folch extraction.

#### 2.2.2. Phospholipid Analysis

Since there is little information in the literature on chia seed phospholipid composition, phospholipid composition was analyzed before and after solvent removal using high performance liquid chromatography with mass spectrometry (LC-MS).

Analysis revealed that total phospholipid content in chia seed oil was 7.49% before solvent removal and 0.26% in the liquid phase of the lipid fraction after solvent evaporation. This confirms the significant loss of phospholipids in oil following this step, most likely found in the residue.

Six phospholipid classes were identified. In the sample before evaporation, PL composition was revealed to be 30.87% phosphatidylinositol (PI), 20.20% phosphatidic acid, 19.15% phosphatidylethanolamine (PE), 12.66% phosphatidylglycerol (PG), 11.82% phosphatidylcholine and 5.3% lysophosphatidyl-choline (Lyso-PC).

Data analysis was next performed to determine the FA composition of the chia seed phospholipid classes. This is presented in Table 3 by phospholipid class for the sample before evaporation.

The amount of PL containing PUFA ALA was found to range between 45 and 68% in the different PL classes, with the highest amount found in PI, and the lowest in PC.

### 2.3. Physicochemical Characterization of Nanoemulsions and Nanoliposomes

We next prepared NE from the chia seed oil and phospholipid-rich solid residue, and NL from the phospholipid-rich solid residue. NL and NE were also prepared from rapeseed oil and commercial rapeseed phospholipids, which we have previously characterized [31], as a reference. In general, the average particle size and distribution in the volume of emulsion or suspension depends on the lipid composition and the method of their preparation. The parameters for assessing the quality of NL and NE preparations are the particle sizes and their homogeneity in the total mass. Polydispersity index which is a measure of the heterogeneity of a sample based on size [16,32], size and zeta-potential were determined, and the results are shown in Table 4.

PDI values of all particles were found to be <0.3, indicating monodisperse solutions with controlled size distribution and narrow dispersity [33]. Statistical analysis was performed comparing data obtained for NL and for NE. Size was determined using DLS (dynamic light scattering), and found to be slightly larger than 100 nm, very similar to NL prepared from rapeseed phospholipids. There were no statistically significant differences in either size or charge between chia- or rapeseed-based NL.

On the other hand, the size of chia-based NE was significantly smaller as compared to rapeseed NE and the NE using chia oil and rapeseed PL. However, this difference was not significant when comparing NE using rapeseed PL as emulsifier (rapeseed NE and mixed NE). Interestingly, the PDI was much lower in mixed NE as compared to the other NE particles.

**Figure 2:**
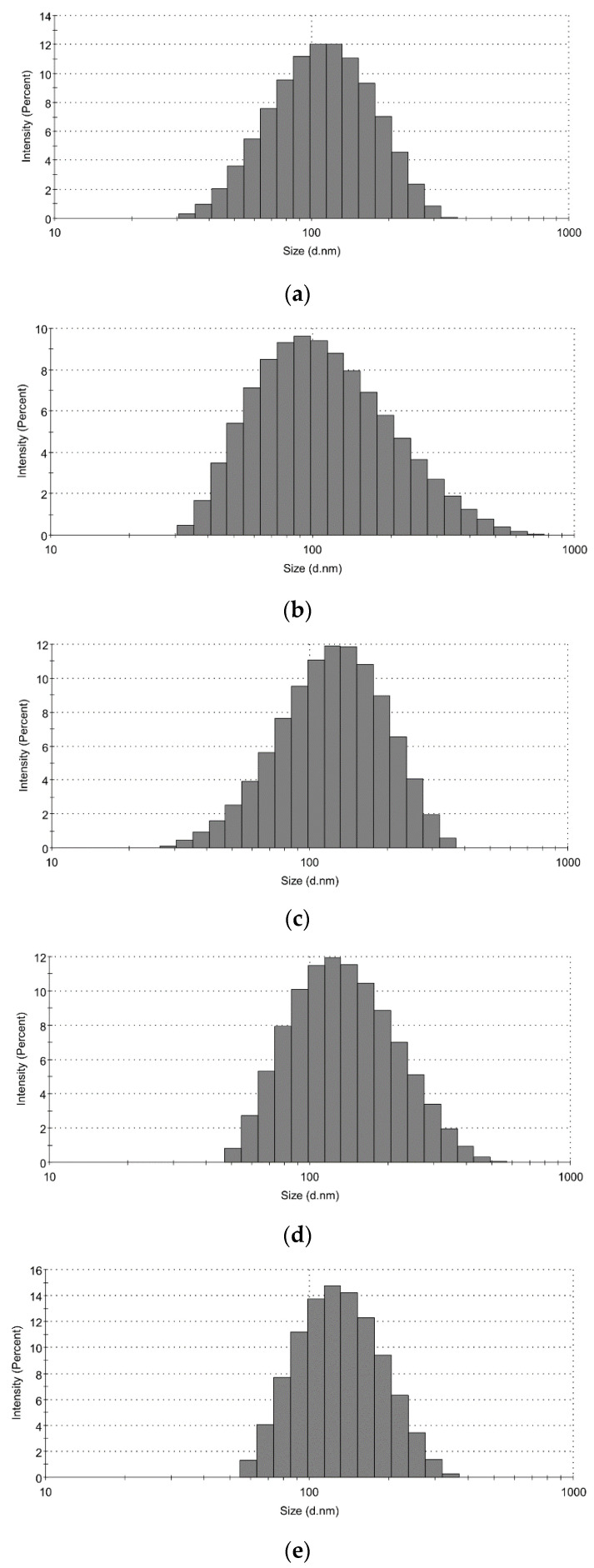
Particle size distribution after preparation: (**a**) Chia NL; (**b**) Rapeseed NL; (**c**) Chia NE; (**d**) Rapeseed NE; (**e**) Mixed NE.

Representative histograms showing size distribution versus intensity of prepared nanoparticles are shown in Figure 2.

We measured the stability of NL and NE by measuring the particle size every day over 5 days after preparation, while stored at 4 °C under N_2_(g) in dark vials. No significant variation was observed during this time period.

**Figure 3:**
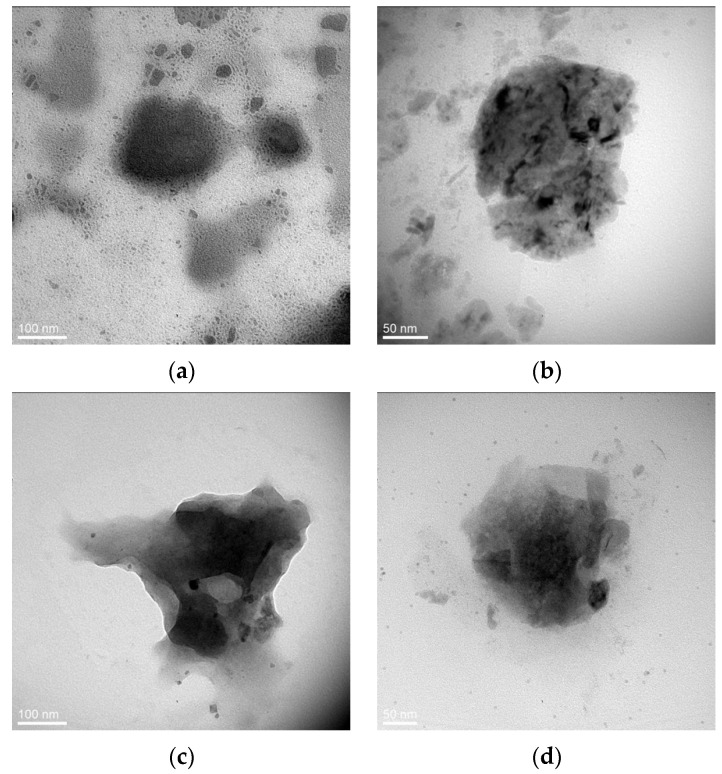
Plant-based nanoparticles by TEM: (**a**) Chia-based NL; (**b**) Chia-based NE; (**c**) Rapeseed-based NL; (**d**) Rapeseed-based NE.

TEM was performed to assess the morphological properties of the particles (Figure 3).

Images showed spherical morphology of chia NL and NE preparations. The structure of the obtained particles was not homogenous, with darker and lighter areas, which suggests the presence of multilayers. Indeed, previous studies in our laboratory, using the similar technique of nanoparticle preparation, revealed the form of multilamellar vesicles because of the sonication step [31].

The rapeseed-based NL appeared slightly larger, but some discrepancies may be expected since the DLS results reflect the hydrodynamic size of the particle, whereas TEM is performed in vacuo.

Finally, we sought to verify the presence of phospholipids in chia NL and NE preparations. Analysis using an enzymatic kit revealed a content of 3.4 ± 1.04 mg/mL and 3.8 ± 1.26 mg/mL choline-containing phospholipids in NL and NE, respectively, confirming the presence of phospholipids in the residue used to prepare these nanoparticles.

## 3. Discussion

In this study, we report the composition of chia seed lipids including phospholipids, and the use of chia oil and phospholipid-rich residue for the preparation of NL and NE. Here, we used chia seeds of the ORURO variety that is cultivated in France. Since the composition of chia seeds, including oil yield and PUFA content, depends on the climate and region of origin, fatty acid composition was determined, and showed a low n-6/n-3 fatty acid ratio. The only study on this variety reported differences in omega-6 and omega-3 content depending on the maturity of the chia grain. Levels of PUFA increased from 74 to 80% up to fruit development, with a similar trend for omega-3, while those of omega-6 PUFA, although higher in earlier stages, decreased when the fruit was close to full maturity [34]. This study therefore demonstrates the interest in using chia seeds before maturation, allowing the recovery of lipid fraction with a low n-6/n-3 fatty acid ratio, which is in agreement with the results obtained here.

The type of solvent extraction method used can affect oil yield from chia seeds ORURO variety, as well as fatty acid composition. Extraction with organic solvents is still one of the most common methods for extracting vegetable oils. Even if oil obtained by cold or hot pressing is considered to be of the highest quality (especially with cold pressing), the main difficulty from the point of view of industrial production is the low oil yield. Therefore, extraction with organic solvents remains one of the preferred methods [35].

A higher yield of chia seed oil was obtained using the Soxhlet apparatus and a mixture of ethers similar to those described previously using petroleum ether [10], as compared to the chloroform and methanol mixture used in the Folch-based procedure [8]. Nevertheless, oil yields with both methods ranged between 29% and 35%, which are comparable to those values provided by the EU commission (between 30% and 35%, [5]). We chose to continue the Folch-based extraction, which is the preferred method for extraction of polar lipids, including phospholipids needed for the preparation of NL. Indeed, little information is available on the use of chloroform-methanol mixture for the extraction of chia seed oil [8,36,37]. Although the Bligh-Dyer method can also be used for polar lipid extraction [38], it is less effective when the sample contains more than 2% lipids as can be found in chia or other oily seeds [39].

Chia oil fatty acid composition reported here for the ORURO variety using the Folch method is similar to those reported by other investigators using other solvents. In general, the literature review shows that the major FA in chia seed oil from different growing regions are ALA (up to 65%), linoleic (up to 20%), palmitic (about 7%), oleic (about 5–7%), and stearic (about 3–4%) acids. Higher ALA content (65.4%) as compared to this study was reported using Soxhlet extraction in petroleum ether of a genotype G8 of chia seeds originating from America and grown in southern Italy [10]. In the study on the chia ORURO variety [34], oil yield from chia by extraction with cyclohexane was slightly lower as compared to the results of this study, ranging from 23.3 to 28.4% from seeds at different stages of development. The content of ALA, according to the authors, varied from 52.4 to 60%, linoleic acid from 19.9 to 22.0%, oleic acid from 0.9 to 11.3%, palmitic acid from 8.2 to 9.2%, and stearic acid from 3.4 to 4%. In our study of chia ORURO, even if the solvent extraction method was different, the profile of MUFA and PUFA is very close to seeds at early stages of maturation.

LC-MS analysis revealed 7.5% phospholipid content in the oil after Folch extraction, which was higher than the values of total polar lipid content obtained using thin-layer chromatography (Iatroscan). This difference may be due to lower sensitivity and detection limit of the latter method, which could contribute to large variations observed following analysis by Iatroscan (for example, we obtained values between 1.86% and 6.94% of polar lipids from an individual sample). Chia PL composition analysis revealed six major PL classes including PI > PA = PE > PG = PC > lyso-PC. The same classes were also found by Calvo et al. (2020) [40] in chia seeds (Mexican origin), with the exception of lyso-PC. Using pressurized liquid or supercritical fluid extraction methods with ethanol as co-solvent, they reported a chia PL yield varying from 0.1% to 16.5%. Recovery was method-dependent, since higher PC levels were obtained following supercritical fluid extraction, while similar amounts of PC and PI were found using pressurized liquid extraction.

Iatroscan and LC-MS analyses revealed a significant loss of polar lipids in the oil after evaporation, which we postulated to be in the solid residue that formed following evaporation of the chlofororm-methanol solvent after lipid extraction. The presence of PC and lyso-PC in NL and NE prepared from the residue was confirmed by analyses using an enzymatic kit for detection of these two choline-containing PL. Our results also showed that this residue was a complex of not only lipids, but also moisture, protein, and dietary fiber, with glucose and fructose only being minor components. Similar solid residues were observed after evaporation in the flax and rapeseeds, and we suggest that these residues may be due to the presence a wide range of possible compounds, including polysaccharides and polyphenols associated with the plant cell membranes [41], leading to the formation of complexes with the phospholipids difficult to re-solubilize after evaporation.

Our results show that chia seed oil and the phospholipid-rich solid residue can be used to prepare stable NL and NE. Indeed, we have previously used PL from other agroresources, including salmon, rapeseed or soya, to prepare stable nanoparticles at a temperature of either 4 °C or 37 °C [31,33,42]. The amphiphilic nature of phospholipids provides the means to prepare stable multilamellar liposomes. Physicochemical characterization revealed both NL and NE particle type to be stable, with a low PDI (<0.3). In drug delivery applications using lipid-based carriers, such as liposome and nanoliposome formulations, a PDI of 0.3 and below is considered to be acceptable and indicates a homogenous population of phospholipid vesicles [16]. The stability of the NL and NE was also demonstrated by their low negative charge (zeta-potential). High value negative/positive charges of these systems are more stable due to repulsive forces. TEM revealed that chia NL and NE were in the 100 nm range, consistent with that measured by DLS, and appeared spherical. Since these particles are PL-based, we would suggest that they are multilamellar in nature, as has been previously demonstrated for other PL-based NL [31]. There is little information in the literature on the use of chia lipid fractions in nanotechnology [15,27]. Here, we show the possibility of preparing chia NE using only chia-derived lipids and PL residue as emulsifier. Stable NE, stored at 4 °C or ambient temperature, have been reported using chia oil, but using exogenous emulsifiers (Tween 80, Span 80 and others) [15]. However, no information has been found in the literature concerning the preparation of NL from chia seed lipids. This is therefore the first report on the preparation and characterization of NL and NE from pure chia seed compounds.

## 4. Materials and Methods

We chose to study the ORURO variety, which is a new genotype bred in France in 2017, adapted to the climatic conditions of the region [34]. ORURO chia seeds (*Salvia hispanica* L.) were purchased from the producer Agrofün, Occitania, France.

Purified rapeseed lecithin (Solae Europe SA society, Geneva, Switzerland), rapeseeds (NATUR-Haus Jurgen Kaiser, NK-Wiebelskirchen, Germany) and flax seeds (*Linum usitatissimum* L.) (Graines de Folie, La E Penell, Normandy, France) were used as for oil extractions (seeds) or NL preparation (rapeseed lecithin).

Ammonium molybdate, internal standard nonadecanoic acid (C19), boron trifluoride diethyl etherate and methanol were purchased from Sigma Aldrich (Saint-Quentin Fallavier, France). Formic acid was obtained from Fluka (Charlotte, NC, USA). Chloroform, hexane, diethyl and petroleum ethers were purchased from Biosolve (Dieuze, France).

### 4.1. Chia Seed Lipids Solvent Extraction

Seed samples were ground before lipid extraction using a portable grinder (Bosch MKM 6003, BSH Electroménager, Saint-Ouen, France).

#### 4.1.1. Soxhlet Method

Extraction of samples (10 g) was carried out using 300 mL of diethyl ether/petroleum ether mixture (1:1) in Soxhlet apparatus (VWR international, Fontenay-sous-Bois, France) for 6 h. The solvent was removed using BÜCHI Rotavapor R-144 rotary evaporator (BÜCHI Sarl, Villebon-sur-Yvette, France) at 40 °C. The vacuum was interrupted by introducing N_2_(g) (inert atmosphere) to avoid oxidative damage to lipids caused by exposure to air.

#### 4.1.2. Folch-Based Method

Lipids were extracted using the modified Folch method (1957) [43]. Chloroform:methanol mixture (600 mL, 2:1, *v/v*) was added to ground chia seeds (100 g) and incubated for 30 min under constant stirring at room temperature. The resulting mixture was vacuum filtered through a funnel with a No. 4 Schott filter, while washing the resulting defatted cake with an additional amount (300 mL) of chloroform:methanol (2:1). The filtered lipid extract was added to a pre-weighed round-bottomed flask, and the solvent was removed using BÜCHI Rotavapor R-144 rotary evaporator (BÜCHISarl, Villebon-sur-Yvette, France) at 50 °C until the solvent was completely removed. The vacuum was interrupted by introducing N_2_(g) (inert atmosphere).

The residue remaining as deposits on the flask wall was collected with a spatula after the removal of the oil fraction. Oil and residue yields were calculated and expressed based on the initial mass of seeds and lipid mass, respectively.

### 4.2. Characterization of Chia Seed Lipid Fraction

#### 4.2.1. Fatty Acid Composition and Characterization of Lipid Classes

Fatty acid composition and lipid classes were characterized as described previously [31,33,42,44].

Briefly, fatty acid methyl esters (FAMEs) were prepared by the Ackman method (1998). Nonadecanoic acid (19:0) (1 mg/mL) was added as an internal standard for the quantitation of fatty acids. The separation of FAMEs was carried out by gas chromatography on a Shimadzu GS 2010-Plus (Perichrom, Saulx-lès-Chartreux, France) equipped with a flame-ionization detector. A fused silica capillary column was used (60 m, 0.25 mm i.d. × 0.2 µm film thicknesses, SP^TM^2380 Supelco, Bellfonte, PA, USA). The column temperature was set initially at 120 °C for 3 min, then increased to 180 °C at a rate of 2 °C min^−1^ and maintained at 220 °C for 25 min. Injection and detection temperatures were set at 250 °C. Standard mixtures (PUFA1 from marine source and PUFA2 from vegetable source; Supelco, Sigma–Aldrich, Bellefonte, PA, USA) were used to identify fatty acids in the elution profile. The results are shown as mean of triplicate determinations.

Lipid classes of chia seed, rapeseed and flax seed extracts were determined by thin layer chromatography (Iatroscan MK-5 TLC-FID, Iatron Laboratories Inc., Tokyo, Japan). Samples (1 µL) were applied onto Chromarod S-III silica coated quartz rods. Lipids were separated by migration in hexane:diethyl ether:formic acid (80:20:0.2, *v*:*v*:*v*) for 25 min, oven dried for 1 min at 100 °C and then scanned in the Iatroscan analyzer. The Iatroscan settings were set with a hydrogen flow rate of 160 mL min^−1^ and air flow rate of 2 L min^−1^. The recording and integration of the peaks were obtained using ChromStar software.

#### 4.2.2. Phospholipid Analysis

Qualitative and semi-quantitative analyses of chia seeds phospholipids were performed on an HPLC-MS system consisting of an UltiMate 3000^TM^ quaternary solvent delivery pump connected to a linear ion trap mass spectrometer (LTQ) equipped with an atmospheric pressure ionization interface operating in electrospray negative ion mode (ESI^-^)(ThermoFisher Scientific, San Jose, CA, USA). Extracts (16 µL) were injected on a LiChroCART (250 mm × 4 mm–5 µm) LiChrospher 100 DIOL column (Merck, Darmstadt, Germany). The flow rate was set at 300 µL·min^−1^ and the column temperature at 30 °C. Mobile phase A consisted of methanol with 0.1% (*v/v*) formic acid, ammonia added to pH 5.3 (approx. 0.05%, *v/v* of ammonia) and 0.05% (*v/v*) triethylamine; mobile phase B was chloroform. Lipids were eluted using a first linear gradient from 95% to 70% of B for 11 min, a second linear gradient to 20% of B for 3 min and an isocratic step at 20% B for 4 min. Mass spectrometric conditions were as follows for ESI^−^ mode: spray voltage was set at −4.5 kV; source gases were set (in arbitrary units min^−1^) for sheath gas, auxiliary gas and sweep gas at 40, 5 and 5, respectively; capillary temperature was set at 230 °C; capillary voltage at −36 V; tube lens, split lens and front lens voltages at −133 V, 70 V and 6.25 V, respectively. Ion optics parameters were optimized by automatic tuning using a standard solution of a phospholipid mixture at 0.1 g·L^−1^ infused in mobile phase (A/B: 5/95) at a flow rate of 5 µL·min^−1^.

Full scan MS spectra (500 to 2000 *m/z*) allowed us to detect parent ions of general form [M − H]^−^ for all the phospholipids of interest except for PC and lyso-PC classes for which the parent ion is [M + HCOO]^−^ due to ionic complex formation between choline group and formiate.

Data dependent MS^2^ and MS^3^ scans were carried out automatically in order to obtain fatty acid composition for phospholipids classes PI, PE, PA, PG, PC and lyso-PC. In the case of PC and lyso-PC phospholipids, MS^3^ scans were necessary to obtain the same level of information because MS^2^ events were only informative of their class (through characteristic neutral loss of 60 corresponding to the simultaneous departure of formiate and a methyl group of choline).

A semi-quantitative analysis of the samples was carried out for each class of phospholipids using MS screening by grouping together the main species identified in the class.

Two phospholipid standard solutions were used (1 g/L). PC and PG were selected as standards. Since the phospholipids PG, PA, PI and PE give rise to a parent ion [M − H]^−^ of similar structure, we estimated that the MS response of these four classes was similar and we used the standard phospholipid PG for their semi-quantitative analysis. The same approach was used for the phospholipids PC and lyso-PC classes, both giving rise to a similar parent ion of structure [M + HCOO]^−^ due to the presence of the choline group. The standard phospholipid PC was therefore used for semi-quantitative analysis of these phospholipids.

Table 5 shows the results of HPLC-MS analysis of standards used to carry out a semi-quantitative analysis of data for all classes of phospholipids present in the lipid fraction of chia seeds ORURO variety.

For the quantitative assessment of the phospholipids mass fraction in the total lipid content of chia seeds, a standard peak area of 13,000,000 units was set.

Since the first sample was used for analysis before removing the solvent, its concentration was estimated based on the mass of chia seed oil obtained after solvent evaporation and the solvent volume used for extraction.

The mass resolution was 0.2 Da. Raw data were processed using the XCALIBUR software program (version 2.1, ThermoFisher Scientific, San Jose, CA, USA).

### 4.3. Nanoparticles Preparation

Nanoparticles were prepared as described by Arab-Tehrany et al. [31] and Hasan [45] with slight modifications.

For nanoemulsions (NE), a 10% (*w/v*) oil-in-water stock solution was prepared. A blend of oil with phospholipids as an emulsifier was used (0.233 g of phospholipids or residue and 0.766 g of oil and 10 mL distilled water). For nanoliposomes (NL), a 2% stock solution (*w/v*) was prepared (0.2 g of phospholipids or residue and 10 mL distilled water). Suspensions were mixed for 4 h under agitation under N_2_(g). The mixtures were then sonicated at 20 kHz at 40% amplitude for 4 min (1 s on and 1 s off) on ice to obtain homogeneous solutions. Samples were stored in sterile 50 mL plastic tubes under N_2_(g) in the dark at 4 °C.

### 4.4. Nanoparticles Size, Polydispersity Index and Zeta-Potential Measurements

The size, polydispersity index (PDI), and zeta-potential of nanoparticles were measured by DLS (Malvern Zetasizer Nano ZS, UK) [33]. Samples were diluted (1:200) using distilled water and then filtered (0.22 µm). Samples were analyzed in standard capillary electrophoresis cells to measure their zeta-potential. Size and zeta-potential measurements were studied with an absorbance of 0.01, a fixed scattering angle of 173°, a refractive index of 1.4650 (for rapeseed-based particles) or 1.4804 (for chia-based particles). Analyses were performed on samples at 25 °C.

### 4.5. Transmission Electron Microscopy

Transmission electron microscopy (TEM) was performed to monitor the microstructure of the nanoparticle samples using negative staining as previously described [31,42]. The samples were diluted 30-fold with distilled water. An equal volume of 2% ammonium molybdate solution was added to the diluted sample, followed by incubation for 3 min at room temperature. A drop of this solution was placed on a carbon face of EMS CF200-CU type carbon-copper grid (200 mesh). The excess liquid was removed by filter paper, and the grid was left to dry (room temperature, 5 min). Micrographs were taken using a Philips/FEI CM200 transmission electron microscope (ThermoFisher Scientific, Mérignac, France) operating at 200 kV and recorded using an Olympus TEM CCD camera.

### 4.6. Stability of Nanoparticules

The aliquots of diluted and filtered nanoparticles prepared at the first day were stored at 4 °C. Mean particle size, charge and PDI of all formulations were analyzed every day during the first 5 days. The same protocol described above (Section 4.4) was used for each analysis.

### 4.7. Phospholipid Determination

Choline-containing phospholipid content of NL and NE were determined using an enzymatic kit (LabAssay™ Phospholipids, Fujifilm Wako Chemicals, Neuss, Germany) according to manufacturer’s instructions.

### 4.8. Statistical Analysis

Statistical analyses were carried out using the Statview^®^ 4.5 statistical package (Abacus Concepts, Inc., Piscataway, NJ, USA).

Comparison of fatty acid composition according to extraction methods were analyzed using Student’s t-test. For nanoparticles characterization, particles were compared using a parametric analysis of variance (ANOVA), considering the type of seeds as the main factor. Post hoc analyses were performed using Fisher’s test when the one-way ANOVAs were significant.

Results are shown as means (± SEM) and statistical significance was set at *p* < 0.05.

## 5. Conclusions

Here, we report for the first time fatty acid composition, phospholipid classes and the phospholipid fatty acid profile of chia seeds of the ORURO variety following Folch extraction. We also demonstrate that stable NE and NL can be obtained from chia oil or chia phospholipid-rich residue fractions using a simple method that involves spontaneous emulsification during lipid hydration. Indeed, such an NL and NE preparation method is a low-energy procedure and is inexpensive, requiring a single sonication step, which can be advantageous for industrial-level production [15,31]. Furthermore, the use of the phospholipid-rich residues for the preparation of NL and NE allows the transformation of a by-product following lipid extraction into potentially useful products as vesicles. While we used a solvent-based method in this initial study to extract chia lipids for composition analysis, chia lipids obtained using sustainable “green” lipid extraction methods merit investigation, which would be of particular interest in ensuring the compatibility of chia-based NE and NL, particularly in applications concerning nutrition and health. In conclusion, we propose that chia lipids represent an interesting source of omega-3 PUFA and phospholipids that can be used in developing NL and NE as nanocarriers for numerous heath-based applications including cosmetics and pharmaceuticals.

## Figures and Tables

**Table 1:** Comparison of fatty acid composition of chia seeds lipid fraction obtained by Soxhlet or Folch extraction methods.

Index	Soxhlet Extraction, %	Folch-Based Extraction, %
C16:0	7.77 ± 0.16	7.28 ± 0.07 *
C18:0	3.56 ± 0.09	3.36 ± 0.01
SFA	11.33	10.64
C16:1	0.24 ± 5.8 × 10^−5^	0.24 ± 0.003
C18:1 n-9	6.87 ± 0.01	6.77 ± 0.03 *
C18:1 n-7	0.79 ± 0.01	0.82 ± 0.01
C18:1 n-5	0.14 ± 0.01	0.13 ± 0.01
MUFA	8.04	7.96
C18:2 n-6	19.16 ± 0.05	18.99 ± 0.06
C18:3 n-6	0.27 ± 0.01	0.25 ± 0.001
C18:3 n-4	0.27 ± 0.004	0.26 ± 0.003 *
C18:3 n-3	60.94 ± 0.20	61.92 ± 0.16 *
PUFA	80.64	81.42
SFA/MUFA	1.41	1.34
SFA/PUFA	0.14	0.13
Ratio n-6/n-3	0.32	0.31

* *p* < 0.05 (Student’s *t*-test); Soxhlet extraction (*n* = 3), Folch extraction (*n* = 3).

**Table 2:** Polar lipid content before and after solvent removal in three seed types by Folch-based extraction.

Seed Type	Before Solvent Evaporation	After Solvent Evaporation
	% total lipids	
Chia seed lipids	4.57 ± 0.69	2.21 ± 0.33
Flax seed lipids	3.70 ± 0.31	2.25 ± 0.53
Rapeseed lipids	3.00 ± 0.70	1.55 ± 0.58

**Table 3:** Molecular species composition of chia seeds PL determined by liquid chromatography with mass spectrometry (LC-MS).

FA Fragments	PA	PI	PG	PE	PC	Lyso-PC
	Relative composition (%)					
16:0	-	-	-	-	-	12.68
16:0/18:2	15.09	21.50	24.07	22.56	19.05	-
16:0/18:3	13.73	32.57	20.57	15.40	13.35	-
18:0	-	-	-	-	-	17.18
18:0/18:2 + 18:1/18:1	-	-	-	6.65	-	-
18:0/18:2	-	9.98	-	-	6.89	-
18:0/18:3	5.40	20.52	6.33	4.47	4.55	-
18:1/18:1 + 18:0/18:2	6.19	-	7.46	-	-	-
18:1/18:2	5.40	-	4.59	5.68	5.13	-
18:1/18:3	4.24	-	3.36	-	3.91	-
18:2	-	-	-	-	-	38.76
18:2/18:2	15.03	-	10.64	19.02	11.13	-
18:2/18:3	23.35	9.49	15.63	19.15	15.80	-
18:3	-	-	-	-	-	31.37
18:3/18:3	11.57	5.94	7.35	7.07	7.48	-
n/a	-	-	-	-	6.23	-
n/a	-	-	-	-	6.49	-

**Table 4:** Characterization of nanoparticles after preparation.

Source	Particle	PDI	Size, nm	Zeta-Potential. mV
Chia	NL	0.208 ± 0.02	117.65 ± 5.88	−31.775 ± 3.88
Rapeseed	NL	0.214 ± 0.01	116.85 ± 2.96	−28.95 ± 2.76
Chia	NE	0.205 ± 0.01	103.80 ± 2.70	−34.10 ± 7.23
Rapeseed	NE	0.194 ± 0.02	131.90 ± 4.69 *	−30.075 ± 2.39
Chia/rapeseed	Mixed NE	0.138 ± 0.01 *	127.30 ± 1.50 *	−27.33 ± 0.58

* *p* < 0.05 (Fisher’s test), for each NL or NE (*n* = 4), mixed NE (*n* = 3).

**Table 5:** Results of chromatographic analysis of phospholipid standards.

Standard, 1 g/L	Retention Time, min	MA Square, Arbitrary Units	M (Molecular Mass), g·mol
Phosphatidylcholine (PC)	7.45	13,345,558	760
Phosphatidylglycerol (PG)	9.89	13,075,849	749
